# Metformin and Risk of Malignant Brain Tumors in Patients with Type 2 Diabetes Mellitus

**DOI:** 10.3390/biom11081226

**Published:** 2021-08-17

**Authors:** Chin-Hsiao Tseng

**Affiliations:** 1 Department of Internal Medicine, National Taiwan University College of Medicine, Taipei 10051, Taiwan; ccktsh@ms6.hinet.net; 2 Division of Endocrinology and Metabolism, Department of Internal Medicine, National Taiwan University Hospital, Taipei 10051, Taiwan; 3 Division of Environmental Health and Occupational Medicine, National Health Research Institutes, Zhunan 350, Taiwan

**Keywords:** diabetes mellitus, malignant brain tumors, metformin, Taiwan

## Abstract

The risk of malignant brain tumors associated with metformin use has rarely been investigated in humans. This retrospective cohort study investigated such an association. Patients with new-onset type 2 diabetes mellitus diagnosed from 1999 to 2005 in the nationwide database of Taiwan’s national health insurance were used to enroll study subjects. We first identified an unmatched cohort of 153,429 ever users and 16,222 never users of metformin. A cohort of 16,222 ever users and 16,222 never users matched on propensity score was then created from this unmatched cohort. All patients were followed up from 1 January 2006 until 31 December 2011. The incidence density was calculated and hazard ratios were derived from Cox regression incorporated with the inverse probability of treatment weighting using a propensity score. The results showed that 27 never users and 155 ever users developed malignant brain tumors in the unmatched cohort. The incidence rate was 37.11 per 100,000 person-years in never users and 21.39 per 100,000 person-years in ever users. The overall hazard ratio comparing ever users versus never users was 0.574 (95% confidence interval: 0.381–0.863). The respective hazard ratios comparing the first (<27.13 months), second (27.13–58.33 months), and third (>58.33 months) tertiles of cumulative duration of metformin therapy versus never users were 0.897 (0.567–1.421), 0.623 (0.395–0.984), and 0.316 (0.192–0.518). In the matched cohort, the overall hazard ratio was 0.317 (0.149–0.673) and the respective hazard ratios were 0.427 (0.129–1.412), 0.509 (0.196–1.322), and 0.087 (0.012–0.639) for the first, second, and third tertile of cumulative duration of metformin therapy. In conclusion, this study shows a risk reduction of malignant brain tumors associated with metformin use in a dose–response pattern. The risk reduction is more remarkable when metformin has been used for approximately 2–5 years.

## 1. Introduction

The incidence of malignant brain tumors (MBT) differs among different countries and glioblastoma is the most common adult primary MBT. A prospective cohort study that followed 8006 Japanese-American men living in Hawaii since 1965 until 1998 showed an incidence of glioblastoma of 6.2 per 100,000 person-years [1]. The age-adjusted incidence was reported to be 3.19 (during 2006–2010), 3.40 (during 2000–2008), 2.05 (during 1999–2003), 0.59 (during 2005), 3.69 (during 2005–2007), and 0.89 (during 2012–2013) per 100,000 population in the USA, Australia, UK, Korea, Greece, and Jordan, respectively [2]. In a recent statistical report derived from the Central Brain Tumor Registry of the United States (CBTRUS), the age-adjusted incidence rate of MBT during 2012–2016 was 7.08 per 100,000 population [3], suggesting an increase in age-adjusted incidence in the USA compared to the rate of 3.19 per 100,000 population during 2006–2010, which was derived from the same dataset of the CBTRUS [4]. In Taiwan, there is a gradual increase in MBT according to a report of the secular trends [5]. For the period from 1980–1984, 1985–1989, 1990–1994, 1995–1999, and 2000–2006, the age-standardized incidence rates per 100,000 population were 1.36, 1.74, 2.29, 2.47, and 2.54, respectively. Men had a higher incidence than women in each specific period, but the increasing trend was observed in both sexes [5].

There is no satisfactory treatment for MBT and the prognosis is poor, with a 5-year survival of <5% after diagnosis for glioblastoma [2]. Some genetic risk factors have been identified for MBT [6,7]. However, occupational and environmental exposures can also increase the risk [8]. These include sugar intake and carbon tetrachloride [1], radiation [9], immune factors and viral interaction [10], and air pollution with long-term exposure to PM2.5 [11].

Metformin is an oral antidiabetic drug that lowers blood glucose and improves insulin resistance. Its major mechanism of glucose-lowering action is by inhibiting hepatic gluconeogenesis through an inhibition of the mitochondrial respiratory chain complex 1. The reduction of hepatic energy status leads to an activation of the 5′ adenosine monophosphate-activated protein kinase (AMPK), a serine/threonine protein kinase [12,13,14]. Beyond glycemic control, metformin exerts a variety of pleiotropic benefits such as weight reduction, improvement of metabolic syndrome, reduction of cardiovascular morbidity, renoprotection, anti-infection, immune-modulation, anti-oxidation, anti-aging, and anti-cancer [14,15].

After oral intake, metformin can cross the blood–brain barrier and distribute to the brain tissues [16,17]. In recent years, the potential usefulness of metformin on the treatment of MBT has been investigated [18]. According to in vitro and in vivo studies, metformin may inhibit the growth of human glioblastoma cells and enhance therapeutic responses to chemotherapy (e.g., temozolomide) and radiotherapy [18,19,20]. Several clinical trials are being conducted to investigate its potential usefulness in the treatment of MBT but the outcomes remain unknown [17,21,22].

Since MBT has very poor prognosis without successful therapeutic modalities, prevention of the disease would be the best way to save lives and to reduce the disease load and economic burden. Whether metformin may exert a protective effect against MBT in humans has rarely been studied. To the best of our knowledge, there was only one previous matched case-control study, which used the UK-based Clinical Practice Research Datalink to investigate the risk of glioma associated with antidiabetic drugs [23]. This study concluded a lack of association between metformin use and glioma [23]. The aim of the present retrospective cohort study was to investigate whether the risk of MBT might be affected by metformin use and whether a dose–response relationship could be demonstrated.

## 2. Materials and Methods

### 2.1. National Health Insurance

This was a retrospective cohort study. The Taiwan’s National Health Insurance (NHI) is a nationwide healthcare system that has been implemented since 1 March 1995. The coverage rate of the NHI is very high and includes 99.6% of the whole population. All in-hospitals and 93% of all medical settings in Taiwan provide medical services under contracts with the Bureau of NHI. Computer files have to be submitted to the Bureau of NHI for reimbursement purpose and these files include information of diagnoses of diseases, prescriptions of drugs, and surgical procedures performed. Academic research proposals using the database can be approved after ethics review. The present study was reviewed and approved by the National Health Research Institutes (number NHIRD-102-175, approved on 5 September 2013). More detail descriptions of the database can be seen in previously published papers [24,25].

### 2.2. Study Population

Diagnoses of diseases in the database were coded by the International Classification of Diseases, Ninth Revision, Clinical Modification (ICD-9-CM) during the whole study period. Accordingly, the codes used in the study for diabetes mellitus were 250 and the code of 191 was used for the diagnosis of MBT.

**Figure 1:**
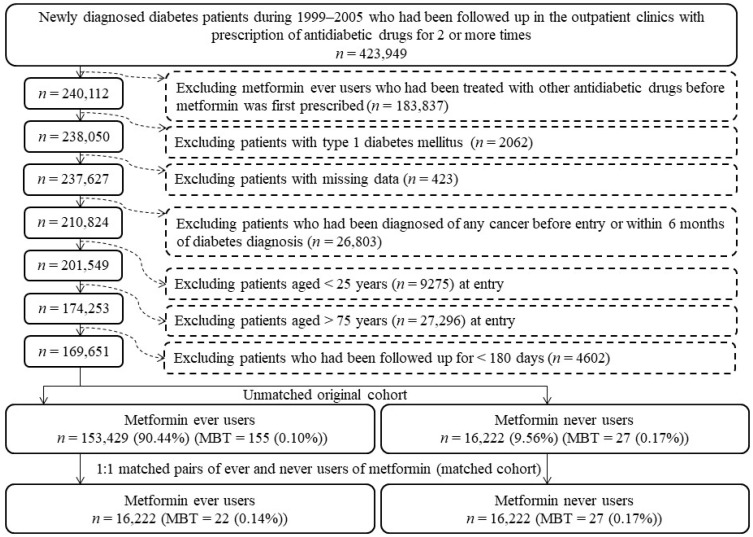
The step-by-step procedures followed to create an unmatched original cohort and a matched cohort from the reimbursement database of the National Health Insurance for the study. The matched pairs of ever users and never users of metformin were derived from the unmatched cohort based on propensity scores (MBT: malignant brain tumors).

An unmatched original cohort and a matched cohort used for analyses in the study were created following the procedures shown in Figure 1 step-by-step. A total of 423,949 patients who had a first diagnosis of diabetes mellitus between 1999 and 2005 and had received outpatient prescriptions of antidiabetic drugs for ≥ 2 times were first identified. Ever users of metformin defined in the study were those who had been prescribed metformin as the first antidiabetic drug without receiving other antidiabetic drugs before the prescription of metformin (*n* = 183,837). Patients with the following conditions were then excluded: (1) patients who were diagnosed of type 1 diabetes mellitus (*n* = 2062), (2) patients who had missing data (*n* = 423), (3) patients who had suffered from any cancer before entry or within 6 months of diabetes diagnosis (*n* = 26,803), (4) patients who were aged < 25 years (*n* = 9275), (5) patients who were aged > 75 years (*n* = 27,296), and (6) patients who had been followed up for a short period of < 180 days (*n* = 4602). As a result, we identified 153,429 ever users and 16,222 never users of metformin and these patients were considered as the unmatched original cohort.

The propensity score (PS) was created by logistic regression that included all characteristics listed in Table 1 and the entry date as independent variables. The Greedy 8 to 1 digit match algorithm using the PS [26] was used to create a cohort of matched pairs of ever users and never users of metformin from the unmatched original cohort. This cohort was considered as the matched cohort.

### 2.3. Potential Confounders

Potential confounders derived from the database and their ICD-9-CM codes are shown in Table 1. These confounders were divided into six categories: (1) demographic and basic data, (2) major comorbidities, (3) diabetes-related complications, (4) antidiabetic drugs, (5) commonly encountered comorbidities, and (6) medications that are commonly used in diabetes patients. Occupations were classified into classes I to IV according to the Bureau of the NHI [27]. Class I included civil servants, teachers, employees of governmental or private businesses, professionals, and technicians. Class II included people without a specific employer, self-employed people, or seamen. Class III included farmers and fishermen. Class IV referred to low-income families supported by social welfare, or veterans.

### 2.4. Statistical Analyses

SAS statistical software, version 9.3 (SAS Institute, Cary, NC, USA) was used to conduct the statistical analyses. Statistical significance was set at a *p* value < 0.05.

The standardized difference between metformin ever users and never users was calculated for each covariate listed in Table 1. The standardized difference is recommended by Austin and Stuart as a test for balance diagnostics [28]. A threshold cutoff value of >10% in standardized difference was used as a potential indicator of imbalance in the variable, which might result in confounding from the variable [28].

The cumulative duration of metformin therapy expressed in months for each patient was calculated from the database. Patients were divided into three subgroups of metformin exposure according to the tertiles of cumulative duration to examine a potential dose–response relationship. The incidence density of MBT was calculated for never users and for users categorized as ever users and as users according to the tertiles of cumulative duration of metformin therapy. The case number of newly identified MBT during follow-up was the numerator of the incidence density. The denominator was expressed as person-years of follow-up. The date of 1 January 2006 was set as the starting date of follow-up. Follow-up ended up to 31 December 2011, at a time when any of the following events occurred first: a new-onset MBT, the date of death, or the date of the last reimbursement record available from the database.

Austin recommended the use of the inverse probability of treatment weighting (IPTW) method to reduce the potential confounding from the differences in covariates [29]. We therefore estimated hazard ratios and their 95% confidence intervals in the unmatched cohort and the matched cohort, respectively, by using Cox regression incorporated with the IPTW using the PS. Hazard ratios were estimated for ever users versus never users, and for ever users, divided by the tertiles of cumulative duration of metformin therapy versus never users.

Sensitivity analyses were then conducted in more restricted patients in the unmatched cohort. First, we excluded patients who had received any two consecutive metformin prescriptions spanning a period of > 4 months. As the prescription of drugs is not allowed to exceed 3 months at each time of prescription, as stipulated by the Bureau of the NHI, we might have excluded most patients with poor adherence when we excluded those patients who did not receive regular drug refills in the analyses. Second, we excluded patients who happened to be treated with incretins during follow-up to prevent potential confounding from the use of incretins (incretin-based therapies were not introduced into the market of Taiwan after the start of follow-up).

## 3. Results

The characteristics in never users of metformin and ever users of metformin and the standardized differences between these two groups of patients are shown in Table 1. In the unmatched original cohort, the values of standardized difference were >10% for age, dyslipidemia, obesity, nephropathy, eye diseases, insulin, meglitinide, acarbose, rosiglitazone, pioglitazone, statin, and fibrate, suggesting potential residual confounding from these variables. However, all values of standardized difference in the matched cohort were <10%, suggesting that the ever and never users of metformin in the matched cohort were well matched for the potential confounders.

Table 2 shows the incidence of MBT in different subgroups categorized according to metformin exposure and the hazard ratios comparing different subgroups of metformin exposure to a referent group of metformin never users. The overall hazard ratios suggested a significantly lower risk in ever users while compared to a referent group of never users. The findings derived from the unmatched cohort and the matched cohort were very similar and a dose–response pattern in terms of the cumulative duration of metformin therapy was observed in the tertile analyses. Metformin use for more than 2 years in the second tertile in the unmatched cohort and more than approximately 5 years in the third tertile in the matched cohort was significantly associated with a reduced risk.

Table 3 shows the sensitivity analyses after excluding patients who had not received regular refill of metformin and after excluding users of incretin-based therapies during follow-up. The results supported the findings in the main analyses shown in Table 2.

## 4. Discussion

### 4.1. Main Findings

The findings of the present study first provide evidence to support that, in patients with type 2 diabetes mellitus, metformin use would be significantly associated with an overall risk reduction of MBT, which could be similarly shown in either the unmatched cohort or the matched cohort in the main analyses (Table 2). A dose–response relationship could be seen in all analyses and a significant risk reduction could be seen when the cumulative duration of metformin therapy was more than 2–5 years (Table 2 and Table 3).

### 4.2. Limitations of an Early Study

The previous matched case-control (1:10) study, which used the UK-based Clinical Practice Research Datalink, included 2005 cases of glioma and 20,050 controls without glioma [23]. The investigators estimated odds ratios of 1.11 (0.59–2.12), 1.42 (0.81–2.47), and 0.72 (0.38–1.39) for patients who received numbers of metformin prescriptions of 1–9, 10–29 and ≥ 30, respectively, in comparison to cases and controls without metformin use [23]. They concluded a lack of significant risk association between MBT and metformin use [23]. However, there are probably some limitations that might have led to the misinterpretation of a lack of effect associated with metformin use in this previous study. First, the investigators included diabetes patients and non-diabetic people in the study and there were only 96 diabetes patients (4.79%) in the cases with glioma (among them: 57 metformin users) and 1240 diabetes patients (6.18%) in the control group without glioma (among them: 716 metformin users) [23]. The numbers of diabetes patients in the cases and controls were actually very small. Second, when they compared the odds ratio for metformin use in each specific subgroup of the number of metformin prescriptions versus non-metformin use, they actually compared metformin users in the diabetes patients versus a group of non-metformin users that was composed mainly of non-diabetic people. Even though the diabetes status had been additionally adjusted for in the models, the small numbers of glioma cases (14, 23, and 20 cases) and the small numbers of metformin users (184, 239, and 350) in the categories of 1–9, 10–29, and ≥ 30 prescriptions of metformin, respectively, might have led to biased estimates because of a lack of sufficient power in the assessment of risk association. Third, in their model, which considered only diabetes patients matched on diabetes duration and A1C level, there were only 86 cases of glioma and 598 controls without glioma. The odds ratios for the numbers of metformin prescriptions of 1–9, 10–29, and ≥30 were 1.07 (0.47–2.40), 1.06 (0.53–2.11), and 0.58 (0.24–1.44), respectively. There seemed to be a neutral effect in the first two subgroups with a lower exposure to metformin but a lower risk could be seen (though not statistically significant) in the third subgroup that had been exposed to a higher cumulative dose of metformin. Similarly, the small number in each diabetes subgroup might have led to a biased conclusion because of a lack of statistical power.

### 4.3. Potential Mechanisms

The mechanisms of the potential protective effect of metformin on MBT remain unknown, but some biological effects of metformin can explain such an observation. MBT cells are dependent on glucose metabolism (a phenomenon known as the Warburg effect) and the mammalian target of rapamycin (mTOR) signaling to support their proliferation and growth [18,30]. Metformin inhibits the mitochondrial complex 1 of electron transport and reduces energy supply to cancer cells [12,18]. Metformin has been shown to inhibit glioblastoma cell growth and induce cell cycle arrest, autophagy, and apoptosis in in vitro studies. These were parallel to an activation of the AMPK and an inhibition of the mTOR pathway and were dependent on genetic and mutational backgrounds [31]. Metformin may also target glioma stem cells, leading to cell cycle arrest and mitochondria-dependent apoptosis [32]. AMPK activation by metformin may also activate the transcriptional activity of p53, a tumor suppressor gene, in MBT cells [18,30]. Lipid peroxidation plays an important role in the development of MBT [33] and MBT cell growth can also be inhibited by reduced fatty acid synthesis and beta-oxidation [30]. Long-term treatment with metformin does inhibit fatty acid synthase and decrease the expression of genes involved in fatty acid oxidation [12]. Leptin, an adipokine that plays important roles in energy balance in the brain, promotes angiogenesis and cell proliferation, survival, and migration via signaling pathways involving the Janus kinase family, signal transducers and activators of transcription, mitogen-activated protein kinase, phosphoinositide-3-kinase, and mTOR, etc. [21]. Metformin can cross the blood–brain barrier and correct these dysregulated pathways of leptin via the activation of AMPK [21]. Ionizing radiation has been linked to the development of MBT in adults and children [9,34]. It is interesting that metformin exerts protection against radiation-induced damage to the lung [35] and skin [36] in animals via the inhibition of inflammatory cytokines.

### 4.4. Implications

First, many ongoing clinical trials are being conducted to investigate the efficacy of metformin as a therapeutic agent for glioblastoma [17,21,22]. The findings of the present study provide some useful clinical information. The requirement of a prolonged use of metformin for more than 2–5 years for a significant preventive effect to be observed (Table 2 and Table 3) implies that a higher dose or even longer duration of therapy may be necessary if metformin is used as a therapeutic agent. However, because of the highly malignant characteristics of most MBTs [2], the usefulness of metformin as a therapeutic agent may be questionable and requires more in-depth investigation.

Second, our previous observational studies conducted in patients with type 2 diabetes mellitus in Taiwan by using the nationwide NHI database suggest that metformin use is associated with a lower risk of various types of cancer, including lung cancer [24], colorectal cancer [25,27], breast cancer [37], thyroid cancer [38], bladder cancer [39], prostate cancer [40], endometrial cancer [41], ovarian cancer [42], cervical cancer [43], kidney cancer [44], oral cancer [45], gastric cancer [46], esophageal cancer [47], nasopharyngeal cancer [48], skin cancer [49], pancreatic cancer [50], hepatocellular cancer [51], biliary tract cancer [52], non-Hodgkin’s lymphoma [53], and bone cancer [54]. These findings suggest that the anti-cancer effects of metformin may involve some common pathophysiological pathways in cancer development, probably targeting many of the hallmarks of cancer [55].

Third, in recent years, some novel antidiabetic drugs such as sodium–glucose cotransporter-2 inhibitors and glucagon-like peptide-1 receptor agonists have been shown to provide cardiovascular and renal protection in patients with type 2 diabetes mellitus [56]. These newer drugs have challenged the positioning of metformin as the first-line therapeutic drug recommended by the American Diabetes Association and the European Association of the Study of Diabetes since 2008 [57]. However, these newer drugs are expensive and their long-term safety, especially with regards to cancer risk, remains unknown. As pointed out by a recent article discussed by Baker et al., up to now, “there are no data to suggest that metformin should not be initiated soon after the diagnosis of diabetes”, even in the era of these novel antidiabetic drugs [56]. Our previous pharmaco-epidemiological studies conducted in Taiwan suggest that metformin not only reduces cancer risk, but also the risk of various non-malignant diseases such as hypertension [58], heart failure [59], atrial fibrillation [60], chronic obstructive pulmonary disease [61], pulmonary tuberculosis infection [62], Helicobacter pylori infection [63], varicose veins [64], acute appendicitis [65], hemorrhoids [66], dementia [67,68], nodular goiter [69], uterine leiomyoma [70], osteoporosis/vertebral fracture [71], and inflammatory bowel disease [72]. We also found that metformin ever users have a lower risk of total mortality than never users of metformin, with an estimated multivariate-adjusted hazard ratio of 0.67 (95% confidence interval: 0.64–0.69) [72]. Therefore, besides the low cost and minimal side effects, the general beneficial effects of metformin on malignant and non-malignant human diseases and on total mortality observed in our previous studies provide a good rationale for using metformin as the first-line therapeutic drug for type 2 diabetes mellitus.

### 4.5. Strengths

Some methodological limitations are commonly seen in pharmaco-epidemiological studies that use big administrative databases to examine the potential clinical outcomes related to medications. In the present study, we carefully addressed these potential limitations to avoid selection bias, prevalent user bias, immortal time bias, and confounding by indication.

The large representative sample derived from the nationwide NHI database is a strong cohort because the database covers 99.6% of the Taiwan’s population. Selection bias could be avoided and the findings can well be generalized to the whole population of Taiwan. To prevent the potential occurrence of prevalent user bias, we purposely included only patients who were newly diagnosed of diabetes mellitus and who were new users of metformin into the study (Figure 1).

Inappropriately assigning the treatment status and follow-up time in the calculation of the follow-up period in the compared subgroups can induce immortal time bias. In the present study, misclassification of the treatment status was not likely by using the universal healthcare system, which keeps all prescription information since 1995, and by enrolling patients who had received ≥2 times of documented prescriptions of antidiabetic drugs (Figure 1). While calculating the follow-up time, we deliberately excluded the immortal time that could happen when the patients were handled with a non-pharmacological approach after diabetes diagnosis (i.e., the time in between diabetes diagnosis and the first antidiabetic drugs prescribed to the patients). Furthermore, we excluded the immortal time that could happen during the initial period of follow-up (i.e., <180 days) (Figure 1). It is worth mentioning that the immortal time that happens within the waiting period between the date of hospital discharge and the dispensing of the drugs prescribed at discharge would not happen in Taiwan. This is because of the fact that in our NHI healthcare system, all discharge drugs of a patient can be obtained immediately from the hospital on the date of the patient’s discharge.

Confounding by indication refers to an association of a risk factor with the indication of a medication under investigation. This can be handled by using a matched cohort of users and non-users balancing in potential confounders [28] and by modeling with Cox regression incorporated with IPTW using PS [29]. The consistency of the findings (Table 2 and Table 3) strengthened a preventive effect of metformin on MBT.

Additionally, there are some other strengths. First, to minimize reverse causality, patients with a diagnosis of MBT within 6 months of diabetes diagnoses had been excluded (Figure 1). Second, the use of pre-existing medical records in the NHI database could reduce the bias resulting from self-reporting. Third, detection bias because of different socioeconomic status could be much reduced because in the NHI healthcare system, the copayments are low and most of them can be waived for low-income patients, veterans, and patients who receive drug refills for chronic diseases.

### 4.6. Limitations

It is acknowledged that there are some study limitations. First, this is a pharmaco-epidemiological study and the basic mechanisms of metformin’s protective effects on the tumorigenesis of MBT could not be investigated.

Second, malignant cells of brain tumors may come from different brain cell types and may have different gene mutations and specifications of metabolic pathways (e.g., glycolytic, glutaminolytic, or oxidative phenotypes) [73]. However, we did not have histopathological data of MBT for disease confirmation and could only use the ICD-9-CM code as a diagnostic tool. The ICD-9-CM code of 191 for MBT diagnosis does not provide detailed information on the histopathology of MBT as classified by the World Health Organization [74].

Third, it should also be noted that the major histopathological types of MBT may differ between adults and children [75,76]. Medulloblastoma is the most commonly seen embryonal tumor in children (approximately 25% of all brain malignancies in children), but it represents < 1% in adults (mainly in young adults < 40 years old) [76,77]. Neuroblastoma is another embryonic cancer involving the sympathetic nervous system that is mainly seen in infants and young children [78]. As metformin is only approved for the treatment of type 2 diabetes mellitus, which is not commonly seen in children, we have excluded patients aged < 25 years in the study (Figure 1). Therefore, the findings of the present study should better not be applied to childhood MBT such as medulloblastoma and neuroblastoma before additional research and confirmation. Neither should the findings be applied to other rare forms of MBT such as ependymoma and sarcoma because they only represent approximately 3% and 2% of MBT, respectively, in Taiwan [79].

Fourth, approximately 80% of MBT arises from glia cells [80] but secondary glioblastomas with genetic mutations in isocitrate dehydrogenase, *TP53* or *ATRX*, etc. are rare [81]. Therefore, primary glioblastoma should be the major category of MBT observed in this study. Metabolic reprogramming has been found to be an important cancer hallmark [82,83] and heterogeneity in the preference of bioenergetic pathways exists in glioblastomas, which can lead to their discrepant sensitivity to metformin [73]. In a recent in vitro study that used three glioblastoma stem cells (GBM18, GBM27, and GBM38) and one human glioblastoma cell line (U87MG) [73], GBM18 seemed to be the most sensitive cell line and GBM27 the most resistant cell line to metformin [73]. GBM18 is characterized by a Warburg-like (glycolytic) metabolism but GBM27 is characterized by a highly oxidative metabolism (up to 50%) with a slower proliferation rate [73]. Such a discrepancy observed in different cell lines was also relative to the effects of metformin on the activation of AMPK, resulting in the inhibition of mTOR [73]. This in vitro study also showed that metformin would affect the survival of normal stem cells less while administered to glioblastoma cancer cells of Warburg-like phenotypes [73]. Therefore, it is reasonable to expect that the effect of metformin on the prevention of MBT might not be the same to all tumors classified under the category of glioblastoma by the World Health Organization [74]. We were not able to answer questions related to such different bioenergetic phenotypes and more in-depth investigation should be tailored to the bioenergetic phenotypes of the tumor cells in future observational studies or interventional clinical trials.

Finally, unmeasured confounders could never be adjusted for and their impacts remained unknown in the study. These potential confounders may include the use of mobile phones, radiation therapy, air pollution, household conditions, education levels, lifestyle, smoking, alcohol drinking, nutritional status, dietary pattern, anthropometric factors, biochemistry, immune profiles, family history, and genetic parameters. However, because a confounder has to be correlated with the exposure (metformin use) and the disease (MBT) at the same time and it must not be in the causal pathway between them [84], there seemed to be no strong evidence to suggest that these unmeasured factors would be correlated with metformin (exposure).

## 5. Conclusions

In summary, this observational retrospective cohort study is the first to suggest a preventive effect exerted by metformin on MBT incidence in patients with type 2 diabetes mellitus. Confirmation with additional consideration of potential confounders, histopathological data, bioenergetic phenotypes, and/or genetic biomarkers is required. Metformin is cheap and safe and its usefulness as a preventive drug or a chemotherapeutic adjuvant to MBT in either diabetes patients or non-diabetic people is worthy of more extensive investigation with prospective cohort designs or randomized controlled clinical trials. Nanotechnology is being applied to develop metformin-derived carbon dots that can more readily cross the blood–brain barrier and accumulate more selectively inside the mitochondria of cancer cells [85]. The application of this novel agent in the treatment of MBT and the tailoring treatment based on metabolic phenotypes of the cancer should provide more promising effects.

## Figures and Tables

**Table 1:** Characteristics of metformin never users and ever users and standardized difference between the two groups.

Variables	Unmatched Cohort					Matched Cohort *				
	Never Users		Ever Users		Standardized Difference	Never Users		Ever Users		Standardized Difference
	(*n* = 16,222)		(*n* = 15,3429)			(*n* = 16,222)		(*n* = 16,222)		
	*n*	%	*n*	%		*n*	%	*n*	%	
Demographic and Basic Data										
Age ** (years)	63.62 ± 10.43		61.84 ± 10.03		−17.75	63.62 ± 10.43		63.83 ± 9.79		2.72
Sex (men)	9298	57.32	82,575	53.82	−7.72	9298	57.32	9238	56.95	−0.98
Occupation										
I	6336	39.06	59,853	39.01		6336	39.06	6359	39.20	
II	3229	19.91	35,286	23.00	8.08	3229	19.91	3177	19.58	−0.81
III	3410	21.02	32,175	20.97	0.07	3410	21.02	3423	21.10	0.36
IV	3247	20.02	26,115	17.02	−8.61	3247	20.02	3263	20.11	0.14
Living Region										
Taipei	5453	33.61	48,388	31.54		5453	33.61	5414	33.37	
Northern	1658	10.22	17,386	11.33	3.74	1658	10.22	1686	10.39	0.59
Central	2841	17.51	28,069	18.29	2.13	2841	17.51	2866	17.67	0.37
Southern	2806	17.30	26,174	17.06	−0.65	2806	17.30	2852	17.58	0.82
Kao-Ping and Eastern	3464	21.35	33,412	21.78	1.22	3464	21.35	3404	20.98	−0.75
Major Comorbidities										
Hypertension (401–405)	13,309	82.04	125,955	82.09	0.23	13,309	82.04	13,315	82.08	0.33
Dyslipidemia (272.0–272.4)	11,723	72.27	127,387	83.03	28.45	11,723	72.27	11,751	72.44	0.72
Obesity (278)	440	2.71	6957	4.53	10.00	440	2.71	411	2.53	−1.13
Diabetes-related Complications										
Nephropathy (580–589)	5666	34.93	42,457	27.67	−17.80	5666	34.93	5557	34.26	−1.80
Eye diseases (250.5, 362.0, 369, 366.41, and 365.44)	3011	18.56	49,861	32.50	32.53	3011	18.56	2854	17.59	−3.07
Stroke (430–438)	5401	33.29	45,899	29.92	−8.11	5401	33.29	5352	32.99	−0.59
Ischemic Heart Disease (410–414)	7773	47.92	70,789	46.14	−3.78	7773	47.92	7800	48.08	0.48
Peripheral arterial disease (250.7, 785.4, 443.81 and 440–448)	3777	23.28	39,982	26.06	6.61	3777	23.28	3688	22.73	−1.42
Antidiabetic drugs										
Insulin	1351	8.33	3571	2.33	−30.61	1351	8.33	1137	7.01	−6.63
Sulfonylurea	11,790	72.68	111,546	72.70	5.66	11,790	72.68	12,199	75.20	5.89
Meglitinide	1340	8.26	6032	3.93	−19.34	1340	8.26	1317	8.12	−0.59
Acarbose	1835	11.31	8397	5.47	−20.71	1835	11.31	1841	11.35	−1.13
Rosiglitazone	479	2.95	7599	4.95	10.83	479	2.95	509	3.14	0.53
Pioglitazone	401	2.47	4049	2.64	−20.71	401	2.47	429	2.64	−1.13
Commonly encountered comorbidities										
Chronic obstructive pulmonary disease (490–496)	8087	49.85	74,987	48.87	−2.40	8087	49.85	8246	50.83	2.11
Tobacco abuse (305.1, 649.0 and 989.84)	460	2.84	6145	4.01	6.67	460	2.84	458	2.82	−0.05
Alcohol-related diagnoses (291, 303, 535.3, 571.0–571.3 and 980.0)	1285	7.92	10,973	7.15	−4.23	1285	7.92	1191	7.34	−2.39
Ocular pterygium (372.40–372.44)	897	5.53	8990	5.86	1.44	897	5.53	894	5.51	0.02
Medications that are commonly used in diabetes patients										
Angiotensin converting enzyme inhibitor/angiotensin receptor blocker	11,298	69.65	112,720	73.47	8.85	11,298	69.65	11,280	69.54	−0.15
Calcium channel blocker	10,215	62.97	92,518	60.30	−5.65	10,215	62.97	10,265	63.28	0.79
Statin	8768	54.05	101,371	66.07	26.41	8768	54.05	8730	53.82	−0.33
Fibrate	5549	34.21	66,521	43.36	20.08	5549	34.21	5474	33.74	−0.81
Aspirin	9333	57.53	95,058	61.96	9.38	9333	57.53	9290	57.27	−0.32

* The matched cohort was created from the unmatched cohort based on propensity score; ** age is denoted by mean ± standard deviation. Refer to Materials and Methods for the classification of occupation. Parentheses include the diagnostic codes of diseases according to the International Classification of Diseases, Ninth Revision, Clinical Modification.

**Table 2:** Incidence rates of malignant brain tumors and hazard ratios by metformin exposure.

Cohort/Metformin Use	Incident Cases of Malignant Brain Tumors	Cases Followed	Person-Years	Incidence Rate (Per 100,000 Person-Years)	Hazard Ratio	95% Confidence Interval	*p* Value
Unmatched Cohort							
Never Users	27	16,222	72,755.38	37.11	1.000		
Ever Users	155	153,429	724,547.50	21.39	0.574	(0.381–0.863)	0.0077
Tertiles of cumulative duration of metformin therapy (months)							
Never Users	27	16,222	72,755.38	37.11	1.000		
<27.13	59	50,605	178,095.31	33.13	0.897	(0.567–1.421)	0.6440
27.13–58.33	58	50,628	248,115.71	23.38	0.623	(0.395–0.984)	0.0426
>58.33	38	52,196	298,336.48	12.74	0.316	(0.192–0.518)	<0.0001
Matched Cohort							
Never Users	27	16,222	72,755.38	37.11	1.000		
Ever Users	9	16,222	76,004.89	11.84	0.317	(0.149–0.673)	0.0028
Tertiles of cumulative duration of metformin therapy (months)							
Never Users	27	16,222	72,755.38	37.11	1.000		
<27.00	3	5343	18,587.13	16.14	0.427	(0.129–1.412)	0.1632
27.00–58.40	5	5361	26,025.97	19.21	0.509	(0.196–1.322)	0.1657
>58.40	1	5518	31,391.79	3.19	0.087	(0.012–0.639)	0.0164

**Table 3:** Sensitivity analyses.

Model/Metformin Use	Incident Cases of Malignant Brain Tumors	Cases Followed	Hazard Ratio	95% Confidence Interval	*p* Value
I. After excluding patients who had received any two consecutive metformin prescriptions spanning a period of four or more months					
Never Users	27	16,222	1.000		
Ever Users	48	51,616	0.568	(0.355–0.911)	0.0188
Tertiles of cumulative duration of metformin therapy (months)					
Never Users	27	16,222	1.000		
<27.13	17	16,879	0.979	(0.527–1.818)	0.9463
27.13–58.33	16	14,014	0.675	(0.363–1.254)	0.2134
>58.33	15	20,723	0.324	(0.172–0.611)	0.0005
II. After excluding patients who happened to be treated with incretins during follow-up					
Never Users	27	15,237	1.000		
Ever Users	151	117,171	0.703	(0.467–1.059)	0.0916
Tertiles of cumulative duration of metformin therapy (months)					
Never Users	27	15,237	1.000		
<27.13	60	42,600	1.002	(0.634–1.582)	0.9940
27.13–58.33	53	38,328	0.710	(0.446–1.128)	0.1471
>58.33	38	36,243	0.428	(0.261–0.703)	0.0008

## Data Availability

The datasets presented in this article are not readily available because public availability of the dataset is restricted by local regulations to protect privacy.
